# Home Exercise Training Improves Exercise Capacity in Cirrhosis Patients: Role of Exercise Adherence

**DOI:** 10.1038/s41598-017-18320-y

**Published:** 2018-01-08

**Authors:** Calvin Kruger, Margaret L. McNeely, Robert J. Bailey, Milad Yavari, Juan G. Abraldes, Michelle Carbonneau, Kim Newnham, Vanessa DenHeyer, Mang Ma, Richard Thompson, Ian Paterson, Mark J. Haykowsky, Puneeta Tandon

**Affiliations:** 1 Cirrhosis Care Clinic, Department of Medicine, University of Alberta, Edmonton, AB Canada; 2 Faculty of Rehabilitation Medicine, University of Alberta, Edmonton, AB Canada; 3 Liver Health Clinic, Royal Alexandra Hospital, Edmonton, AB Canada; 4 Department of Biomedical Engineering, University of Alberta, Edmonton, AB Canada; 5 Division of Cardiology, Department of Medicine, University of Alberta, Edmonton, AB Canada; 6 College of Nursing and Health Innovation, University of Texas at Arlington, Arlington, Texas USA

## Abstract

Cirrhosis patients have reduced peak aerobic power (peak VO_2_) that is associated with reduced survival. Supervised exercise training increases exercise tolerance. The effect of home-based exercise training (HET) in cirrhosis is unknown. The objective was to evaluate the safety and efficacy of 8 weeks of HET on peak VO_2_, 6-minute walk distance (6MWD), muscle mass, and quality of life in cirrhosis. Random assignment to 8 weeks of HET (moderate to high intensity cycling exercise, 3 days/week) or usual care. Exercise adherence defined as completing ≥80% training sessions. Paired t-tests and analysis of covariance used for comparisons. Forty patients enrolled: 58% male, mean age 57 y, 70% Child Pugh-A. Between group increases in peak VO_2_ (1.7, 95% CI: −0.33 to 3.7 ml/kg/min, p = 0.09) and 6MWD (33.7, 95% CI: 5.1 to 62.4 m, p = 0.02) were greater after HET versus usual care. Improvements even more marked in adherent subjects for peak VO_2_ (2.8, 95% CI: 0.5–5.2 mL/kg/min, p = 0.02) and 6MWD (46.4, 95% CI: 12.4–80.5 m, p = 0.009). No adverse events occurred during testing or HET. Eight weeks of HET is a safe and effective intervention to improve exercise capacity in cirrhosis, with maximal benefits occurring in those who complete ≥80% of the program.

## Introduction

In addition to the well-known health complications that arise with liver dysfunction^1^, it is increasingly recognized that many patients with cirrhosis also have severe physical deconditioning, sarcopenia, and physical frailty^2–5^. These prevalent, interwoven conditions limit normal daily activities, increase the risks for pre-transplant death and removal from liver transplant wait lists^2,3,6^, and lengthen hospital stays post-transplantation^7–11^.

The peak aerobic power, as measured by the peak VO_2,_ is the most objective and integrated measure of impaired exercise capacity; an increase of 3.5 mL/kg/min is associated with a survival advantage in patients referred for cardiac testing^12^. Abnormalities in the peak VO_2_ have been independently linked to the severity of hepatic dysfunction across the cirrhosis patient spectrum. Even patients with well compensated, early stage cirrhosis, have an approximately 40% lower peak VO_2_ relative to healthy controls^8,13^.

Unlike most advanced liver-related complications, exercise capacity is potentially modifiable with changes in nutrition and exercise therapy. Our group and others have confirmed that supervised exercise training significantly improves peak VO_2_, muscle mass, and fatigue, and reduces the hepatic venous pressure gradient in patients with Child-Pugh A or B cirrhosis^14–16^.

Although clinically advantageous, the delivery of exercise using a supervised, site-based training environment does not meet the needs of many patients with cirrhosis. Individually, patients report many barriers to participation, including issues with transportation, parking availability, out-of-pocket costs, poor health, and interference with domestic responsibilities as well as social and work obligations^17,18^.

Home-based exercise training (HET) is an alternative delivery modality that has several advantages. It is less biased in its potential inclusion of highly motivated patients and simulates a program that can be continued in the long-term. Moreover, home-based programs are often more cost-effective than site-based ones, flexible with the timing of exercise sessions, and accessible to all patients. Although data have been discordant, the lack of direct supervision can result in a loss of exercise efficacy^19–21^. To date, there has been no data on the efficacy of home-based exercise interventions in cirrhosis.

We have conducted the largest randomized controlled trial (RCT) in cirrhosis to assess the efficacy of an 8-week HET program on peak VO_2_ (primary outcome), aerobic endurance, muscle mass, quality of life, and safety. We tested the hypothesis that HET would significantly improve peak VO_2_, aerobic endurance, 6MWD, muscle mass, and quality of life without adverse events compared to a usual care (UC) group.

## Results

### Baseline characteristics

**Figure 1:**
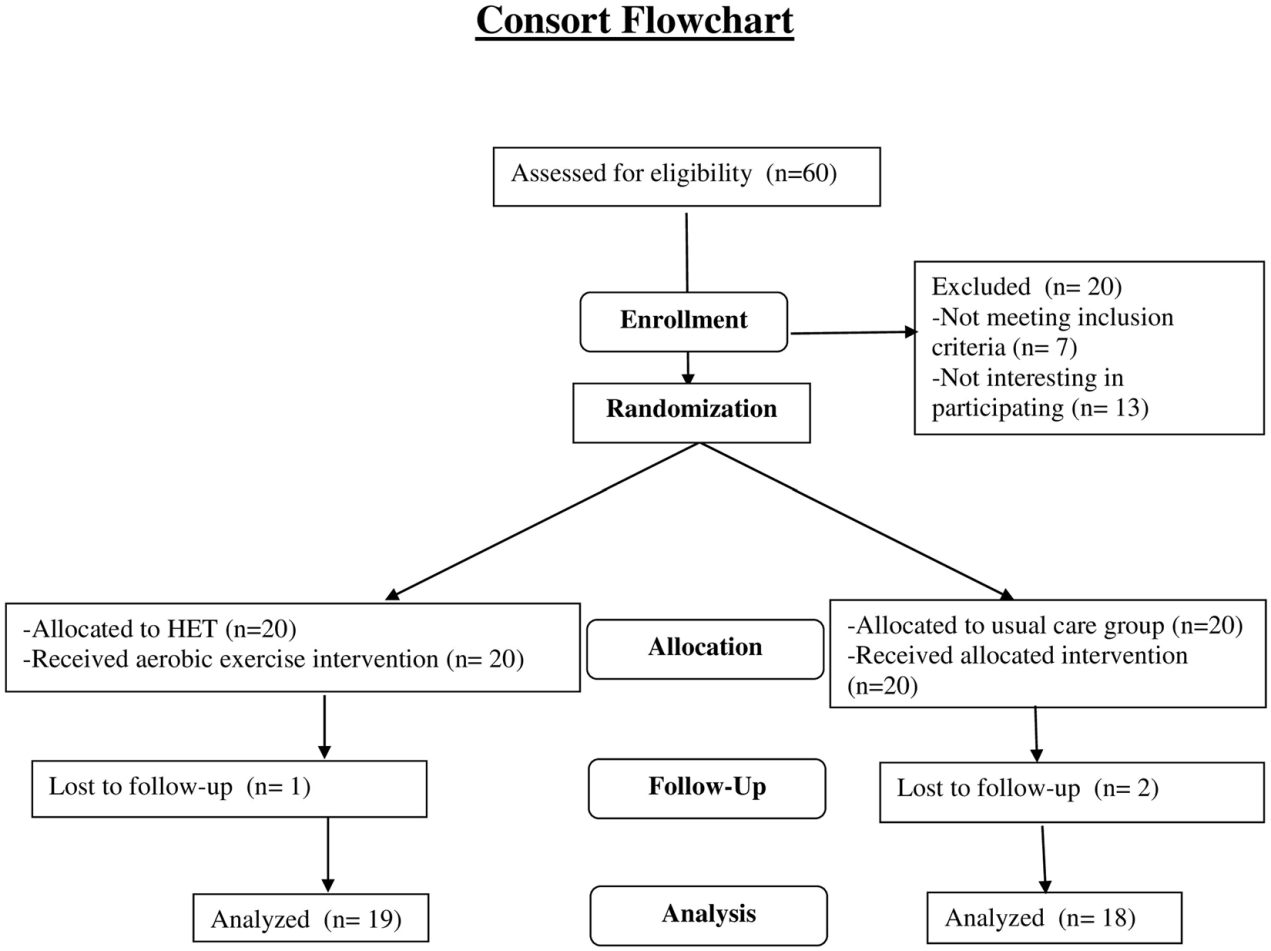
Patient trial flow. The flow of patients through the trial as per CONSORT guidelines.

As shown in Fig. 1, 60 patients were approached at one of two tertiary care liver clinics in Alberta (University of Alberta Hospital and Royal Alexandra Hospital) of which 40 were eligible to participate. Seven patients were excluded because of comorbid illnesses that precluded participation (cardiac n = 2, dialysis n = 1, hepatocellular carcinoma n = 2, and inability to use the exercise bike n = 2). The remaining 13 were not interested in participating.

Baseline characteristics (Table 1) of recruited patients showed that 58% were male, mean age was 57 years, mean MELD was 9.4 ± 2.9, 70% had Child-Pugh A disease and 30% had Child-Pugh B cirrhosis. The majority of patients had either alcohol- or hepatitis C-related cirrhosis. Thirty-five percent of the patients were taking diuretics and 28% met criteria for either primary or secondary esophageal variceal prophylaxis in advance of participating in the RCT. The groups were well-matched apart from a chance occurrence of a significantly lower baseline peak VO_2_ in patients randomized to HET (Table 1).

**Table 1:** Baseline Characteristics.

Characteristic	HET (n = 20)	UC (n = 20)	P value
Age (years)	53.0 ± 8.3	56.4 ± 8.5	0.84
Male gender, n (%)	10 (50)	13 (65)	0.34
Charlson comorbidity index	4.0 ± 1.3	4.1 ± 1.0	0.89
Etiology of cirrhosis, n (%)			
•Alcohol induced	5 (25)	6 (30)	0.9
•NASH	5 (25)	5 (25)	
•Hepatitis C	7 (25)	5 (25)	
•Other	3 (15)	4 (20)	
Severity of liver disease			
•MELD score	9.05	9.70	0.50
•Child-Pugh score	6.35	6.26	0.84
•Child-Pugh A/B (%)	14/6 (70/30)	14/6 (70/30)	0.80
Use of diuretics, n (%)	9 (45)	5 (25)	0.32
History of varices n (%)	14 (70)	15 (75)	0.72
Variceal prophylaxis, n (%)	3 (15)	8 (40)	
•TIPS in place	0 (0)	1 (5)	0.16
•Primary prophylaxis	2 (10)	4 (20)	
•Secondary prophylaxis	1 (5)	3 (15)	
Use of β-blocker, n (%)	3 (15)	5 (25)	0.70
Hemoglobin (g/L)	133.5 ± 15.7	132.5 ± 14.7	0.84

*The etiology of liver disease in the Other category included hepatitis B (n = 3), autoimmune hepatitis (n = 2), primary biliary cirrhosis (n = 1), and primary sclerosing cholangitis (n = 1).

Abbreviations: MELD, Model for End Stage Liver Disease; TIPS, transjugular intrahepatic portosystemic shunt; VO_2_, oxygen uptake.

### Primary Outcome Measure (Peak VO_2_)

**Figure 2:**
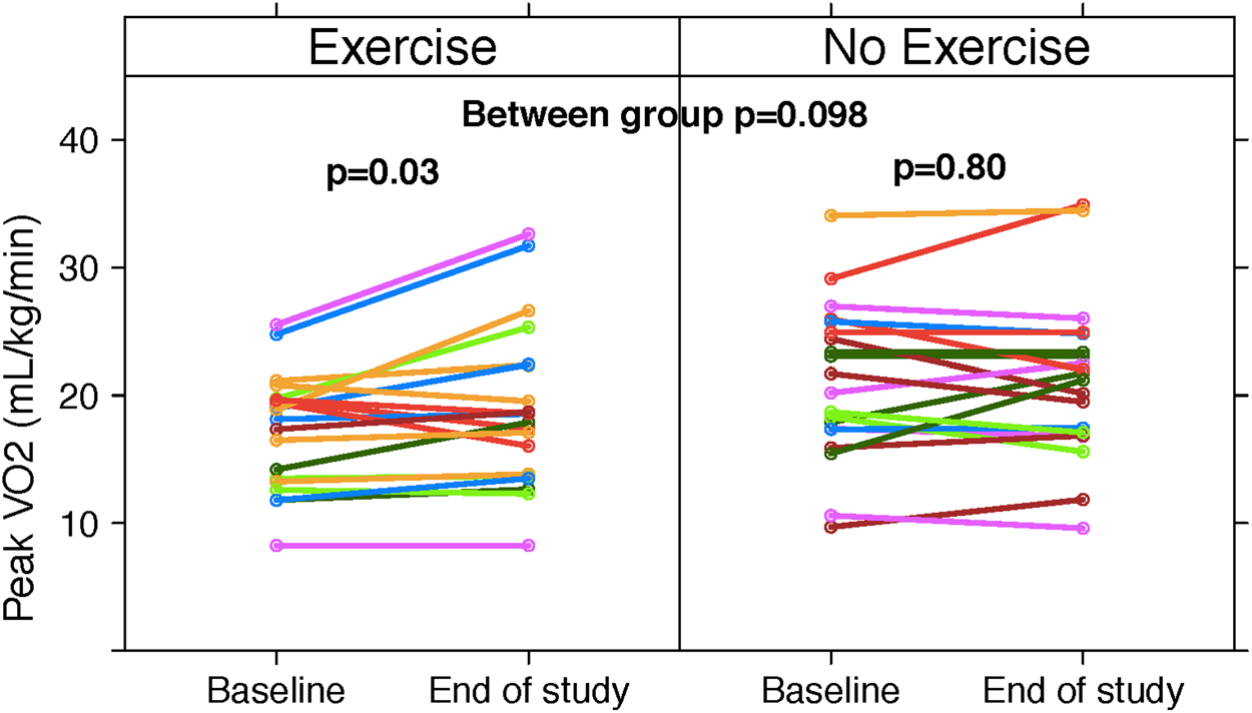
Individual patient changes in Peak VO_2_ (mL/kg/minute). When compared to the UC group, peak VO_2_ in the HET group improved by 1.7 mL/kg/minute (95% CI: −0.33 to 3.7), p = 0.098). Abbreviations: VO_2_, oxygen uptake.

Comparing the changes from baseline to post-intervention, there was a significant increase in peak VO_2_ (L/min and indexed to body mass) within the HET cohort and no change found in UC. The between group comparison of changes in peak VO_2_ did not reach significance (Table 2, Fig. 2

**Table 2:** Baseline and End of Study measurements comparing all patients randomized to exercise (HET) versus usual care (UC).

	**Exercise Training Group (HET)**			**Usual Care Group (UC)**			**Between group mean changes (95% CI)**	**ANCOVA p value between groups**
	**Baseline**	**Study End**	**Within group p-value**	**Baseline**	**Study End**	**Within group p-value**		
**Cardiorespiratory and exercise capacity measures**								
Peak VO_2_ (L/min)	1.44 ± 0.40	1.57 ± 0.56	0.04	1.87 ± 0.66	1.87 ± 0.70	0.98	0.15 (−0.03 to 0.32)	0.095
Peak VO_2_ (mL/kg/min)	17.3 ± 4.5	19.0 ± 6.4	0.03	21.0 ± 6.1	21.2 ± 6.3	0.80	1.7 (−0.33 to 3.7)	0.098
Peak Power output (Watts)	120.0 ± 41.7	125.0 ± 52.2	0.27	149.8 ± 50.9	146.5 ± 59.9	0.47	12.5 (−0.08 to 25.0)	0.051
Peak Heart rate (bpm)	130.4 ± 23.0	126.5 ± 22.4	0.09	139.8 ± 22.3	138.2 ± 22.7	0.72	−4.0 (−11.4 to 3.5)	0.29
Peak Systolic blood pressure (mmHg)	157.4 ± 23.7	150.4 ± 20.0	0.04	157.5 ± 21.0	152.5 ± 16.4	0.007	−0.56 (−7.4 to 6.3)	0.87
6-Minute walk distance (m)	476.0 ± 89.8	490.7 ± 104.1	0.08	520.8 ± 92.2	500.4 ± 91.8	0.08	33.7 (5.1 to 62.4)	0.02
**Anthropometric measures**								
Thigh Circumference (cm)	50.6 ± 5.8	52.4 ± 6.6	0.024	51.2 ± 5.8	51.8 ± 5.5	0.23	1.20 (−0.58 to 2.98)	0.18
Average Feather Index (cm/m^2^)*	1.25 ± 0.40	1.31 ± 0.38	0.05	1.21 ± 0.26	1.27 ± 0.24	0.07	0.005 (−0.08 to 0.09)	0.91
Average Compression Index (cm/m^2^)*	0.75 ± 0.27	0.77 ± 0.25	0.30	0.75 ± 0.18	0.73 ± 0.19	0.72	0.04 (−0.04 to 0.12)	0.33
Body Mass Index (kg/m^2^)	29.3 ± 5.2	29.3 ± 5.1	0.74	28.9 ± 5.1	29.2 ± 5.0	0.90	0.12 (−0.6 to 0.85)	0.73
Body Weight (kg)	84.6 ± 15.6	84.8 ± 14.7	0.85	88.1 ± 16.9	89.1 ± 16.4	0.82	−0.005 (−2.0 to 2.0)	0.99
**Quality of life and liver function parameters**								
EQ-VAS	63.0 ± 16.9	62.6 ± 16.4	0.91	70.4 ± 24.8	72.9 ± 18.5	0.58	7.01 (−16.6 to 2.6)	0.15
CLDQ								
Total	5.06 ± 1.09	4.76 ± 1.16	0.13	5.22 ± 1.36	5.03 ± 1.34	0.29	0.15 (−0.40 to 0.66)	0.57
Abdominal Symptoms	5.43 ± 1.54	5.30 ± 1.43	0.66	4.93 ± 1.82	5.32 ± 1.71	0.06	−0.23 (−0.91 to 0.45)	0.49
Systemic Symptoms	5.01 ± 1.12	4.53 ± 1.36	0.07	5.04 ± 1.51	5.10 ± 1.49	0.72	0.55 (−0.06 to 1.15)	0.07
Activity	5.08 ± 1.42	4.73 ± 1.50	0.20	5.32 ± 1.71	4.77 ± 2.13	0.21	−0.12 (−1.11 to 0.86)	0.80
Emotional Function	5.31 ± 1.04	4.84 ± 1.22	0.03	5.63 ± 1.32	5.46 ± 1.33	0.23	0.34 (−0.14 to 0.82)	0.16
Worry	5.30 ± 1.33	5.18 ± 1.37	0.59	5.42 ± 1.69	5.34 ± 1.75	0.83	0.08 (−0.71 to 0.88)	0.83
Fatigue	4.28 ± 1.53	4.14 ± 1.35	0.55	4.46 ± 1.52	4.16 ± 1.68	0.24	−0.12 (−0.77 to 0.53)	0.72
MELD	9.05 ± 2.61	9.57 ± 2.51	0.47	9.71 ± 3.16	10.3 ± 3.65	0.59	0.35 (−2.02 to 2.73)	0.76
Child-Pugh score	6.35 ± 1.46	6.53 ± 1.46	0.85	6.26 ± 1.24	6.42 ± 1.46	0.72	0.44 (−0.21 to 1.09)	0.18
ALT (units/L)	46.8 ± 38.27	47.19 ± 50.53	0.98	44.69 ± 37.2	40.1 ± 33.3	0.67	−4.3 (−14.2 to 5.6)	0.001
Bilirubin (µmol/L)	23.53 ± 16.3	27.2 ± 20.4	0.65	37.3 ± 51.3	34 ± 40.5	0.83	−3.9 (−18.6 to 10.8)	0.59
Albumin (g/L)	38.1 ± 4.50	37.1 ± 5.27	0.72	37.8 ± 4.44	37.9 ± 4.92	0.95	−0.69 (−3.4 to 2.05)	0.61

Abbreviations: CLDQ, Chronic Liver Disease Questionnaire; EQ-VAS, EQ-Visual Analogue Scale; MELD, Model for End Stage Liver Disease; VO_2_, oxygen uptake.

).

### Secondary Outcome Measures

**Figure 3:**
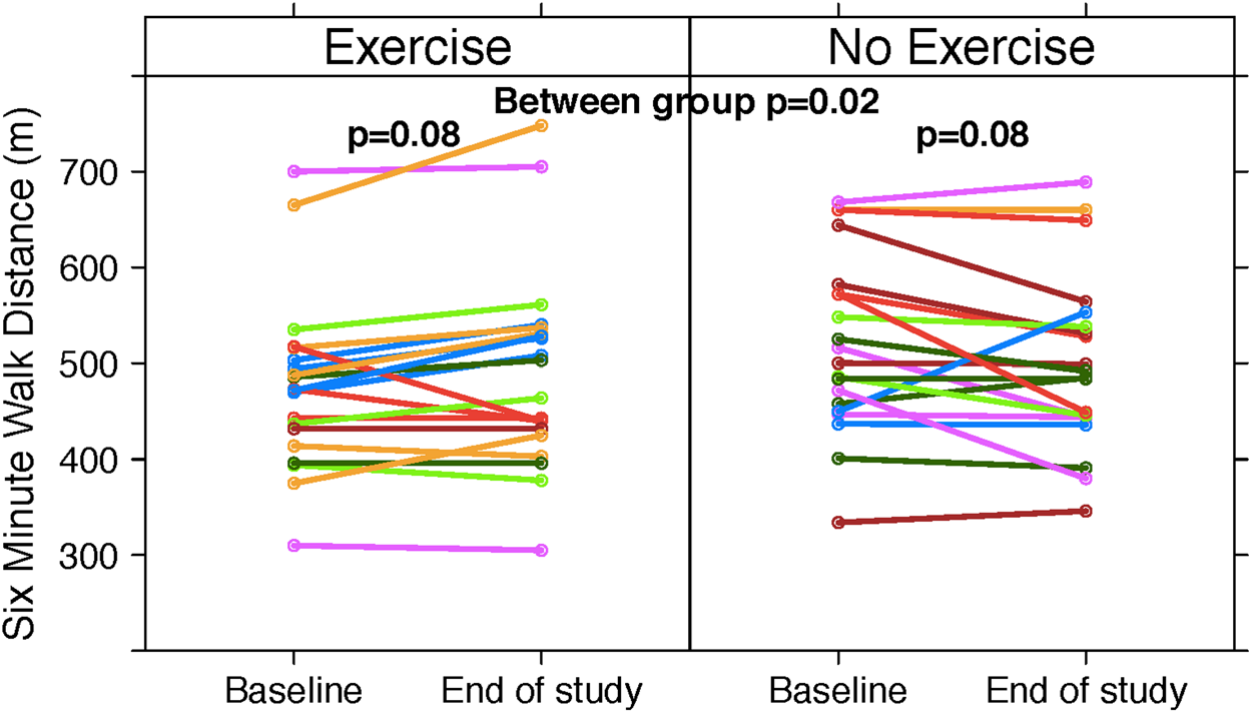
Individual patient changes in 6MWD (meters). When compared to the UC group, 6MWD in the HET group improved by 33 meters (95% CI: 5.1 to 62.4), p = 0.02). Abbreviations: 6MWD, six minute walk distance.

Within the HET patient group, a statistically significant mean increase in aerobic endurance was found using the 6-minute walk distance (6MWD; 33.7 m, 95% CI: 5.1 to 62.4, p = 0.02) (Fig. 3). HET was associated with a significant increase in the thigh circumference (p = 0.02) and a trend to improvement in thigh muscle thickness as measured by the average feather index (p = 0.05) using a portable ultrasound device. In the UC cohort, there were no significant within group changes for these measures. As well, there was no significant between group differences for these outcomes. As for quality of life values, no significant within or between group differences were identified.

### Adherence (sub-group analysis)

Patients in the exercise group were almost equally divided into two groups based on their HET program adherence. Eleven (55%) patients fulfilled criteria for adherence defined as completing ≥80% of the sessions (80% of sessions n = 1, 96% n = 1, and ≥100% n = 9; mean adherence 100% ± 9% of sessions). The remaining 9 (45%) patients were classified as non-adherers (0% of sessions n = 5; 21% n = 2, 38% n = 1, and 66% n = 1; mean adherence 16% ± 23% of sessions) (Table 3). The only significant predictor of exercise adherence was male gender, where 9/11 (82%) adherers and 1/9 (11%) non-adherers were men. Younger age also trended to significance on univariate analysis (p = 0.06) as a predictor of good adherence (Table 4). One patient in the HET group and two patients in the UC group were lost to follow-up and did not complete end of study testing.

**Table 3:** Subgroup analysis - Baseline and End of Study measurements comparing the subgroup of patients who adhered to the exercise regimen (n = 11) versus Usual Care (n = 20).

	Exercise Training Group Adhered to ≥80% of sessions (n = 11)^*^			Usual Care (UC) Group (n = 20)			Between group mean changes (95% CI)	ANCOVA p value between groups
	Baseline	Study End	Within group p-value	Baseline	Study End	Within group p-value		
Peak VO_2_ (mL/kg/min)	18.5 ± 4.5	21.4 ± 6.7	0.01	21.0 ± 6.1	21.2 ± 6.3	0.80	2.8 (0.5 to 5.2)	0.02
6-Minute walk distance (m)	503.6 ± 96.3	534.0 ± 102.7	0.003	520.8 ± 92.2	500.4 ± 91.8	0.08	46.4 (12.4 to 80.5)	0.009

^*^% of prescribed sessions completed: Mean 100% ± 9.0% (SD); Median 100% (IQR: 100 to 104%).

**No significant differences in thigh muscle circumference, muscle mass or quality of life.

**Table 4:** Predictors of adherence amongst the exercise training group.

Predictors	OR (95% CI)	P value
Male gender^*^	36.0 (2.7 to 476.3)	0.007
Age	0.86 (0.74 to 1.01)	0.058
Beta-blocker use	1.78 (0.13 to 23.5)	0.66
Etiology of liver disease		
•Hepatitis C	1	Reference
•Alcohol	0.5 (0.05 to 5.15)	0.56
•NASH/Cryptogenic	1.13 (0.11 to 11.6)	0.92
•Other	1.5 (0.09 to 25.4)	0.78
BMI baseline	0.98 (0.82 to 1.17)	0.83
MELD score at baseline	2.8 (0.8 to 9.8)	0.11
Six minute walk distance at baseline	1.0 (0.996 to 1.03)	0.16
VO_2_ baseline (mL/kg/min)	1.16 (0.93 to 1.46)	0.19
EQ-VAS baseline	0.97 (0.92 to 1.03)	0.38
CLDQ total score baseline	1.80 (0.72 to 4.55)	0.21
CLDQ fatigue subscore baseline	0.91 (0.50 to 1.65)	0.75

^*^Of the 20 patients randomized to the exercise therapy group, in the “non-adherent” group, 8/9 were women as compared to only 2/11 in the adherent group.

### Adverse events

HET was well tolerated by all 20 patients and no adverse events (e.g., musculoskeletal injuries, falls, variceal bleeding, worsening liver function) occurred during exercise testing or training (Table 2).

## Discussion

Based upon the largest RCT conducted to date in a cirrhosis setting, we found that an 8-week unsupervised home exercise program (HET) did not significantly increase peak aerobic power (peak VO_2_) relative to the control group (UC) according to our primary between group analysis. However, increases in both aerobic endurance (6MWD) and thigh muscle thickness for the HET cohort were significantly greater than the UC. Moreover, sub-analysis within HET found that patients completing ≥80% of the sessions (mean adherence of 100% ± 9% of sessions) achieved both clinically and statistically significant improvements for peak VO_2_ and aerobic endurance (6MWD) compared to those with a low mean adherence of 16% ± 23% of sessions.

Patients with cirrhosis have severe and marked exercise intolerance. A recent systematic review of 1107 cirrhotic patients, undergoing screening for liver transplantation^11^, reported a mean peak VO_2_ of 17.4 mL/kg/minute, a value typically indicative of a sedentary female aged 80–89 years^22,23^. Consistent with this finding, the mean baseline peak VO_2_ of the exercise-trained participants included in the current trial was 17.3 mL/kg/minute. This value corresponds to significant functional limitation as it is below the VO_2_ threshold level (e.g., 18 mL/kg/minute) required for full and independent living^22,23^.

The primary study endpoint, peak VO_2_ only trended to significance using the primary analysis of between group effects. Importantly though, clinical and statistically significant improvements were seen based upon within exercise group analyses and also when the results were analyzed in the subgroup of patients who were adherent to the exercise regimen. Why was the between group change in the peak VO_2_ with HET versus UC in the current trial lower than that achieved in our prior supervised exercise training trial^16^, despite a similar population and exercise protocol? This may be due to several factors. Specifically, some UC subjects began regular exercise (despite us asking them to continue with their regular activity). Moreover, patients in the HET group had a statistically lower baseline VO_2_ than those in the UC group. As demonstrated in Supplemental Fig. 1, a higher baseline VO_2_, was associated with a higher likelihood of improving with exercise therapy, possibly disadvantaging our HET group. Lastly, and perhaps most importantly, as with pharmacological therapy, the benefit from exercise is strongly related to adherence to the prescribed regimen. Notably, adherence in the current study was significantly lower than in our earlier supervised site base exercise study^16^. Sub-analysis by exercise adherence in the current study, revealed that with adherence, the peak VO_2_ increased by 2.8 mL/kg/min a value very close to the 3.5 mL/kg/min improvement in fitness that is associated with a survival advantage in patients referred for cardiac testing^12^. Similar to the dose response seen with pharmacological therapy, exercise has the potential to show significant benefits if patients can adhere to the prescribed regimen. In this study, adherence defined by completion of at least 80% of the sessions.

The 6 minute walk test independently predicts mortality in patients with cirrhosis who are awaiting liver transplantation^24^. As compared to the original study by Carey *et al*., the mean 6MWD in our study participants was reasonably well preserved, at 498 m. Short-term exercise interventions have resulted in improvements in the 6-minute walk distance in other clinical populations, including those with heart failure^25^. In keeping with this, we noted a significant between group improvement in aerobic endurance (33.7 m), which increased even more when just the participants with “good” adherence were considered (46.4 m). These 6MWD improvements are in the range of the 20 to 50 meters associated with clinically meaningful improvements in functional performance status, including a patient’s ability to more easily carry out activities of daily living^26,27^.

This study was subject to several limitations, the first being the small sample size. Our power calculations were done using an estimated value for improvement in the primary outcome based on the improvement seen in Zenith *et al*.^16^. Studies using a larger sample size may demonstrate a more definitive benefit. Secondly, because of the limitations set during our inclusion criteria, these results are generalizable only to a predominantly compensated population of cirrhosis patients. Seventy percent of patients included in the study had Child-Pugh class A disease. It was considered most appropriate to begin the evaluation of HET in a predominantly compensated population as there is limited data on exercise in general in cirrhosis and as HET has not previously been evaluated in these patients. Future studies can extend this to patients with decompensated disease. Thirdly, beta-blocker therapy was not an exclusion criterion, and a small but comparable number of patients were on beta-blockade in each group. Data from the cardiac rehabilitation literature suggests that although beta-blockers may blunt the improvement in the peak VO_2_^28^, training effects and prognostic information can still be achieved despite therapeutic doses. Beta-blocker use did not affect our primary outcome.

Adherence rates were lower than seen in our supervised trial^16^ and played a major role in the overall efficacy of the intervention. The reduced adherence may in part have been related to the home-based exercise setting. Although home-based training has the advantage of flexibility and the lack of the need to travel in for sessions, it lacks the camaraderie and formal discipline of training in a group or in a hospital based setting. This may compromise the participant’s ability to exercise at a given intensity for long periods of time. Future studies may choose to address this challenge by altering the way home based training is performed, including the tailoring of the regimen by choice of different exercises based on patient preferences and patient identified barriers to exercise^29–31^. Adherence toolkits^32^, choosing activities that are seen to have an immediate purpose (e.g., walking for transportation), that are associated with social engagement and that participants are confident performing^33^ can also improve adherence. Although not well studied in cirrhosis, resistance training has been shown to be a potent stimulus for reversing muscle wasting and improving exercise capacity across a range of other populations^34–36^. In our experience, this exercise modality may be better tolerated and induce less fatigue than prolonged aerobic exercise in chronically ill and deconditioned populations^37,38^ and deserves further evaluation in cirrhosis. Additionally, given the “digital age”, the use of remote monitoring and check in systems as well as video conference training sessions may help to re-establish the camaraderie and make HET more into an enjoyable and accountable “group” activity which can be carried out in the comfort of the patient’s home. Data in other populations have associated e-health physical activity programs^39–42^ with improvements in clinical outcomes, quality of life and reduced health care utilization. The only independent predictor of good adherence in our study was male gender, with younger age trending to significance. Across populations, there is discordant data about how age and gender influence adherence in the literature^43–46^. Notably, there has been no systematic evaluation of the specific predictors of adherence in cirrhosis. Future studies should consider more detailed evaluation of this as well as targeted reduction of sex-specific barriers in order to optimize adherence^31^.

Despite the noted study limitations, this is the largest RCT of exercise therapy in cirrhosis and adds to accumulating data in the area^14,16^ regarding the safety of exercise training as well as the importance of adherence to the regimen.

In conclusion, these findings indicate that 8 weeks of HET is an effective therapy to improve peak aerobic power, submaximal aerobic endurance and thigh muscle mass in clinically stable Child-Pugh class A and B cirrhosis. Maximal benefits are seen in patients with adherence to the prescribed regimen. Future studies are needed to confirm our findings of efficacy and importantly of safety in early cirrhosis, to extend these evaluations to patients with more advanced disease and to determine if measures targeted to simulating the “group training” appeal of supervised training can increase adherence in those patients undergoing HET. Recognizing the challenges that many patients have with traveling to site-specific exercise facilities, the important prognostic implications of physical deconditioning and the limited alternative options available for treatment, with behavioural change modifications to improve adherence, we see HET as moving one step closer to expanding exercise as a routine part of the therapeutic armamentarium in cirrhosis.

## Methods

This randomized controlled pilot study was conducted at the University of Alberta Hospital in Edmonton, Alberta, Canada, from October 2014 to June 2015. All potentially eligible patients were consecutively screened during clinic visits at the Cirrhosis Care Clinic and all provided signed informed consent. Study approval was granted by our local Health Research Ethics Board (University of Alberta, protocol number Pro00048610) and the study was registered at ClinicalTrials.gov (Study ID: NCT02267421, date of registration October 14, 2014). All methods were performed in accordance with relevant ethics guidelines and regulations. All co-authors had access to the study data and reviewed and approved the final manuscript.

### Patient Selection

Inclusion criteria were: (1) cirrhosis diagnosed by compatible radiological appearance or biopsy, (2) Child-Pugh class A (CP-A) or B (CP-B), (3) age ≥18 and ≤70 years, and (4) guideline-based primary prophylaxis in place for high risk gastroesophageal varices (either non-selective beta-blockade or endoscopic band ligation to the point of variceal eradication)^47^. Subjects were excluded if they had: (1) significant cardiac disease (ejection fraction <60% or history of coronary artery disease, positive exercise stress test (≥1 mm ST segment depression)), (2) chronic renal failure on dialysis, (3) hemoglobin <110 g/L, (4) human immunodeficiency virus infection, (5) hepatocellular carcinoma, (6) active non-hepatocellular carcinoma related malignancy, (7) myopathy, (8) any physical impairment or orthopedic abnormality preventing ET, or (9) post-liver transplantation.

Randomization to either the HET or UC groups was performed using a computer randomization plan (http://www.randomization.com/). Treatment assessments were concealed by sealed envelope. Patients and investigators were informed of the study group assignment after completion of the baseline assessment. The primary outcome was change in peak VO_2_ from baseline. Secondary outcomes included changes in quadriceps muscle thickness as measured by ultrasound, thigh circumference, 6MWD, quality of life, and safety as determined by the occurrence of variceal hemorrhage or deteriorations in liver biochemistry, Child-Pugh, and MELD scores.

Prior to and just after the intervention, the following assessments were performed.

- *Cardiopulmonary (VO*_2_ *peak) exercise testing* was performed on an electrically braked cycle ergometer^48^. A physician and an exercise specialist supervised this test. The initial power output was set at 15 Watts (W) and increased by 15 W every 2 minutes until the aerobic threshold was achieved, then the intensity increased by 15 W every minute until volitional exhaustion. Continuous expired gas analysis was performed with a metabolic measurement system (Innocor, Innovision, Denmark). Blood pressure and heart rate were monitored and the highest oxygen consumed over a one-minute period was used as the peak VO_2_ score^48^.
- *Muscle Mass and thigh circumference*- A portable ultrasound machine (Mindray^©^, Shenzhen, China) was used to measure the depth of the right quadriceps muscle (the rectus femoris and vastus intermedius) as we have previously described^16,49^. Two readings were obtained at each point: a compression reading taken by pressing the probe downwards until no further compression of the muscles was possible and a featherweight reading where the probe was held without pressure on the thigh. Measurements at both points were averaged and corrected for stature (height^2^) to yield average compression and featherweight indices. Thigh circumference was evaluated at the one-third point using a flexible tape measure.
- *6-minute walk distance-* A standardized 6MWD was performed in accordance with American Thoracic Society Guidelines^27^.
- *Quality of life measures - The Chronic Liver Disease Questionnaire (CLDQ)* ^50^ and *EQ-Visual Analogue Scale (EQ-VAS)* ^51^ were used to assess quality of life and self-perceived health status. The CLDQ is a 29-item self-administered Health Related Quality of Life instrument^50^. It includes items in the domains of fatigue, activity, emotional function, abdominal symptoms, systemic symptoms, and worry, rated on a 7-point Likert scale. Higher scores indicate a better health related quality of life^52^. The *EQ-VAS* is a component of the EQ-5D, a standardized measure of health status that quantitatively records a patient’s self-rated health state on a 100-point visual analogue scale^51^. Patients were asked to rate their own health state along the scale with end-points labeled “best imaginable health state” and “worst imaginable health state”. Higher scores indicate a better health status.
- *Lab tests and liver disease severity -* Serum albumin, bilirubin, creatinine, ALT, AST, electrolytes, CBC, and INR were performed. Child-Pugh and MELD were calculated.

### Interventions

After baseline testing, patients were randomized to either HET or UC for 8 weeks.

The HET group was visited at home by an exercise specialist to deliver and set up a cycle ergometer (Schwinn), and provide instruction about the exercise-training program. The exercise intensity was prescribed at a heart rate of 60 to 80% of heart rate reserve, or at 14 to 15 on the 6–20 Borg scale. Exercise training was performed 3 days/week for 30 minutes each session and based on patient tolerance was gradually increased to 60 minutes of exercise per session. Each workout was to be preceded by a 5-minute warm up and concluded with a 5-minute cool down at a lower intensity. Participants were contacted by phone weekly and based on patient availability and interest, attempts were made to visit them biweekly for exercise session observation. Patients in the UC group were not given formal exercise prescription guidelines and were asked to continue their regular activity for the study duration. All patients were asked to complete an activity diary daily to assess adherence. This diary was reviewed by the exercise specialist on the weekly phone visits and at the end of the study. Exercise adherence was defined by participation in ≥80% of prescribed exercise sessions^53^. In addition to assessing adherence, during the weekly phone calls made by the exercise specialist, patients were also asked about adverse events (musculoskeletal injuries, falls, worsening liver function, hospitalization).

At the baseline assessment, a registered dietician (VM) provided nutritional counseling to all patients. Optimal protein and calorie targets were based on the European Society of Enteral and Parenteral Nutrition guidelines^54^ and the *Nutrition Support Manual (Adult)* and *Daily Nutrient Recommendations for Liver Disease* provided by Alberta Health Services. For protein intake, 1.2–1.5 g/kg protein was recommended in all patients daily. With BMI >30, protein was dosed based on an ideal body weight (BMI 24.9). Daily target calorie intake was BMI specific ranging from 35–40 kcal/kg for a BMI of 20–30, 25–35 kcal/kg for a BMI of 30–40 and 20–25 kcal/kg for a BMI of >40^55^. On exercise days, patients were asked to consume an additional 250–300 kcal to replete the calories burned with exertion and account for their impaired capacity to mobilize glucose during exercise^8^.

The datasets generated during and/or analysed during the current study are available from the corresponding author on reasonable request.

### Statistical Analysis

The sample size calculation was based on peak VO_2_. Based on our prior supervised exercise intervention trial^16^, we expected the improvement in peak VO_2_ to be 5.3 ml/kg/min greater after HET compared to UC. With a between group standard deviation of 5 ml/kg/min^16^, alpha level p < 0.05 and power of 80%, a total of 28 subjects were needed. Given the potential for a combination of dropouts and for decreased efficacy in HET versus a supervised exercise environment, we conservatively increased the target recruitment by 40% to a total of 40 subjects (n = 20 exercise, and n = 20 controls).

Statistical analysis was performed using SPSS version 19 (SPSS Inc., Chicago, IL, USA). Variables were described using means and standard deviations, or proportions. To take advantage of the RCT design, the primary statistical analysis was the determination of between-group differences using the analysis of covariance (ANCOVA). ANCOVA was chosen as it accounts for the individual changes within patients, changes between groups relative to baseline values, and is robust to violations of normality assumptions^56^. We also assessed within-group differences (e.g., before and after within each of the UC and HET groups) using paired t-tests. Statistical significance was established at a 2-tailed p-value of <0.05. Finally, we also evaluated the impact of exercise adherence compared to controls using ANCOVA methodology.

## Electronic supplementary material

Supplementary Material

## References

[1] D’Amico G, Garcia-Tsao G, Pagliaro L (2006). Natural history and prognostic indicators of survival in cirrhosis. A systematic review of 118 studies. J Hepatol.

[2] Tandon, P. *et al*. A Rapid Bedside Screen to Predict Unplanned Hospitalization and Death in Outpatients With Cirrhosis: A Prospective Evaluation of the Clinical Frailty Scale. *Am J Gastroenterol*, 10.1038/ajg.2016.303 (2016). 10.1038/ajg.2016.303 27481305

[3] Lai JC (2014). Frailty predicts waitlist mortality in liver transplant candidates. American journal of transplantation: official journal of the American Society of Transplantation and the American Society of Transplant Surgeons.

[4] Montano-Loza AJ (2012). Muscle Wasting Is Associated With Mortality in Patients With Cirrhosis. Clin.Gastroenterol.Hepatol..

[5] Tandon P (2012). Severe muscle depletion in patients on the liver transplant wait list - its prevalence and independent prognostic value. Liver Transpl.

[6] Nathan J, Fuld J (2010). Skeletal muscle dysfunction: a ubiquitous outcome in chronic disease?. Thorax.

[7] Montano-Loza AJ (2014). Severe muscle depletion predicts postoperative length of stay but is not associated with survival after liver transplantation. Liver Transpl.

[8] Lemyze, M., Dharancy, S. & Wallaert, B. Response to exercise in patients with liver cirrhosis: Implications for liver transplantation. *Dig Liver Dis*, 10.1016/j.dld.2012.09.022 (2012). 10.1016/j.dld.2012.09.022 23137795

[9] Neviere RE (2013). Implications of Preoperative Aerobic Capacity and Exercise Oscillatory Ventilation AfterLiver Transplantation. Am J Transplantation.

[10] Bernal W (2013). Aerobic capacity at cardio-pulmonary exercise testing and survival with and without liver transplantation in patients with chronic liver disease. Liver Transpl.

[11] Ney M (2016). Systematic review: pre- and post-operative prognostic value of cardiopulmonary exercise testing in liver transplant candidates. Aliment Pharmacol Ther.

[12] Myers J (2002). Exercise capacity and mortality among men referred for exercise testing. N Engl J Med.

[13] Jones JC, Coombes JS, Macdonald GA (2012). Exercise capacity and muscle strength in patients with cirrhosis. Liver Transpl.

[14] Roman E (2014). Randomized pilot study: effects of an exercise programme and leucine supplementation in patients with cirrhosis. Dig Dis Sci.

[15] Berzigotti, A. *et al*. Effects of an intensive lifestyle intervention program on portal hypertension in patients with cirrhosis and obesity: The sportdiet study. *Hepatology*, 10.1002/hep.28992 (2016). 10.1002/hep.28992 27997989

[16] Zenith, L. *et al*. Eight weeks of exercise training increases aerobic capacity and muscle mass and reduces fatigue in patients with cirrhosis. *Clin Gastroenterol Hepatol* **12**, 1920-1926 e1922, 10.1016/j.cgh.2014.04.016 (2014). 10.1016/j.cgh.2014.04.016 24768811

[17] Doolan-Noble F, Broad J, Riddell T, North D (2004). Cardiac rehabilitation services in New Zealand: access and utilisation. N Z Med J.

[18] Jones M (2007). ‘DNA’ may not mean ‘did not participate’: a qualitative study of reasons for non-adherence at home- and centre-based cardiac rehabilitation. Fam Pract.

[19] Hwang R, Marwick T (2009). Efficacy of home-based exercise programmes for people with chronic heart failure: a meta-analysis. European journal of cardiovascular prevention and rehabilitation: official journal of the European Society of Cardiology, Working Groups on Epidemiology & Prevention and Cardiac Rehabilitation and Exercise Physiology.

[20] Fokkenrood HJ (2013). Supervised exercise therapy versus non-supervised exercise therapy for intermittent claudication. Cochrane Database Syst Rev.

[21] Ashworth NL, Chad KE, Harrison EL, Reeder BA, Marshall SC (2005). Home versus center based physical activity programs in older adults. Cochrane Database Syst Rev, CD004017.

[22] Wilson TM, Tanaka H (2000). Meta-analysis of the age-associated decline in maximal aerobic capacity in men: relation to training status. American journal of physiology. Heart and circulatory physiology.

[23] Fitzgerald MD, Tanaka H, Tran ZV, Seals DR (1997). Age-related declines in maximal aerobic capacity in regularly exercising vs. sedentary women: a meta-analysis. Journal of applied physiology.

[24] Carey EJ (2010). Six-Minute Walk Distance Predicts Mortality in Liver Transplant Candidates. Liver Transpl.

[25] O’Connor CM (2009). Efficacy and safety of exercise training in patients with chronic heart failure: HF-ACTION randomized controlled trial. JAMA.

[26] Perera S, Mody SH, Woodman RC, Studenski SA (2006). Meaningful change and responsiveness in common physical performance measures in older adults. Journal of the American Geriatrics Society.

[27] Brooks D, Solway S, Gibbons WJ (2003). ATS statement on six-minute walk test. Am J Respir Crit Care Med.

[28] Wolfel EE (2006). Exercise testing with concurrent beta-blocker usage: is it useful? What do we learn?. Current heart failure reports.

[29] Ney M (2017). Patient-perceived barriers to lifestyle interventions in cirrhosis. Saudi J Gastroenterol.

[30] Jones WK (2003). Understanding barriers to physical activity is a first step in removing them. American journal of preventive medicine.

[31] White JL, Ransdell LB, Vener J, Flohr JA (2005). Factors related to physical activity adherence in women: review and suggestions for future research. Women Health.

[32] Babatunde FO, MacDermid JC, MacIntyre N (2017). A therapist-focused knowledge translation intervention for improving patient adherence in musculoskeletal physiotherapy practice. Archives of Physiotherapy.

[33] Lee HH, Emerson JA, Williams DM (2016). The Exercise-Affect-Adherence Pathway: An Evolutionary Perspective. Front Psychol.

[34] Sharif S (2011). Resistance exercise reduces skeletal muscle cachexia and improves muscle function in rheumatoid arthritis. Case Rep Med.

[35] Keilani, M. *et al*. Effects of resistance exercise in prostate cancer patients: a meta-analysis. *Supportive care in cancer: official journal of the Multinational Association of Supportive Care in Cancer*, 10.1007/s00520-017-3771-z (2017). 10.1007/s00520-017-3771-z PMC5527087 28600706

[36] Kogure GS (2016). Resistance Exercise Impacts Lean Muscle Mass in Women with Polycystic Ovary Syndrome. Medicine and science in sports and exercise.

[37] McNeely ML, Campbell KL, Courneya KS, Mackey JR (2009). Effect of acute exercise on upper-limb volume in breast cancer survivors: a pilot study. Physiother Can.

[38] Gould DW, Lahart I, Carmichael AR, Koutedakis Y, Metsios GS (2013). Cancer cachexia prevention via physical exercise: molecular mechanisms. J Cachexia Sarcopenia Muscle.

[39] Galiano-Castillo N (2013). Telehealth system (e-CUIDATE) to improve quality of life in breast cancer survivors: rationale and study protocol for a randomized clinical trial. Trials.

[40] Kairy D, Lehoux P, Vincent C, Visintin M (2009). A systematic review of clinical outcomes, clinical process, healthcare utilization and costs associated with telerehabilitation. Disabil Rehabil.

[41] Dlugonski D, Motl RW, McAuley E (2011). Increasing physical activity in multiple sclerosis: replicating Internet intervention effects using objective and self-report outcomes. J Rehabil Res Dev.

[42] Lovo Grona, S. *et al*. Use of videoconferencing for physical therapy in people with musculoskeletal conditions: a systematic review. *J Telemed Telecare*, 1357633X17700781, 10.1177/1357633X17700781 (2017). 10.1177/1357633X17700781 28403669

[43] Dolansky MA, Stepanczuk B, Charvat JM, Moore SM (2010). Women’s and men’s exercise adherence after a cardiac event. Res Gerontol Nurs.

[44] Stiggelbout M, Hopman-Rock M, Crone M, Lechner L, van Mechelen W (2006). Predicting older adults’ maintenance in exercise participation using an integrated social psychological model. Health Educ Res.

[45] Bautista-Castano I, Molina-Cabrillana J, Montoya-Alonso JA, Serra-Majem L (2004). Variables predictive of adherence to diet and physical activity recommendations in the treatment of obesity and overweight, in a group of Spanish subjects. Int J Obes Relat Metab Disord.

[46] Kampshoff CS (2014). Determinants of exercise adherence and maintenance among cancer survivors: a systematic review. Int J Behav Nutr Phys Act.

[47] Garcia-Tsao G, Sanyal AJ, Grace ND, Carey W (2007). Prevention and management of gastroesophageal varices and variceal hemorrhage in cirrhosis. Hepatology..

[48] Haykowsky M, Taylor D, Kim D, Tymchak W (2009). Exercise training improves aerobic capacity and skeletal muscle function in heart transplant recipients. American journal of transplantation: official journal of the American Society of Transplantation and the American Society of Transplant Surgeons.

[49] Tandon, P. *et al*. A Model to Identify Sarcopenia in Patients With Cirrhosis. *Clin Gastroenterol Hepatol*, 10.1016/j.cgh.2016.04.040 (2016). 10.1016/j.cgh.2016.04.040 27189915

[50] Younossi Z, Guyatt G, Kiwi M, King D, Boparai N (1999). Development of a disease-specific health-related quality of life index for chronic liver disease. Gut.

[51] Rabin R, de Charro F (2001). EQ-5D: a measure of health status from the EuroQol Group. Annals of medicine.

[52] Schulz KH, Kroencke S, Ewers H, Schulz H, Younossi ZM (2008). The factorial structure of the Chronic Liver Disease Questionnaire (CLDQ). Quality of life research: an international journal of quality of life aspects of treatment, care and rehabilitation.

[53] Mandic S (2009). Effects of aerobic or aerobic and resistance training on cardiorespiratory and skeletal muscle function in heart failure: a randomized controlled pilot trial. Clinical rehabilitation.

[54] Plauth M (2006). ESPEN Guidelines on Enteral Nutrition: Liver disease. Clin.Nutr..

[55] Amodio P (2013). The nutritional management of hepatic encephalopathy in patients with cirrhosis: International Society for Hepatic Encephalopathy and Nitrogen Metabolism Consensus. Hepatology.

[56] Vickers AJ, Altman DG (2001). Statistics notes: Analysing controlled trials with baseline and follow up measurements. BMJ.

