# Supplementary material for: Home Exercise Training Improves Exercise Capacity in Cirrhosis Patients: Role of Exercise Adherence
Source: Sci Rep. 2018 Jan 8;8:99. doi: 10.1038/s41598-017-18320-y (PMC5758716; doi:10.1038/s41598-017-18320-y)

Title: Home Exercise Training Improves Exercise Capacity in Cirrhosis Patients: Role of Exercise Adherence

Authors: Calvin Kruger[1,2], Margaret L. McNeely[2], Robert J. Bailey[3], Milad Yavari[2], Juan G Abraldes[1], Michelle Carbonneau[1], Kim Newnham[1], Vanessa DenHeyer[1], Mang Ma[1], Richard Thompson[4], Ian Paterson[5], Mark J. Haykowsky[6**], Puneeta Tandon[1,2**]

**These authors contributed equally as senior authors on the manuscript.

Supplementary Figure 1 Legend

Supplementary Fig.1: Plot comparing the before and after peak VO_2 values in patients in the control group and in the exercise (HET) group. Patients in the control group did not show changes in their peak VO_2. In patients in the HET group, higher baseline peak VO_2 values were associated with higher chances of improvement in peak VO_2 at the end of the study (regression lines represent a linear regression model, in which the predictor, baseline peak VO_2, was modelled with cubic splines with 2 knots; grey bands represent the 95% CIs of the predictions).

Supplementary Figure 1

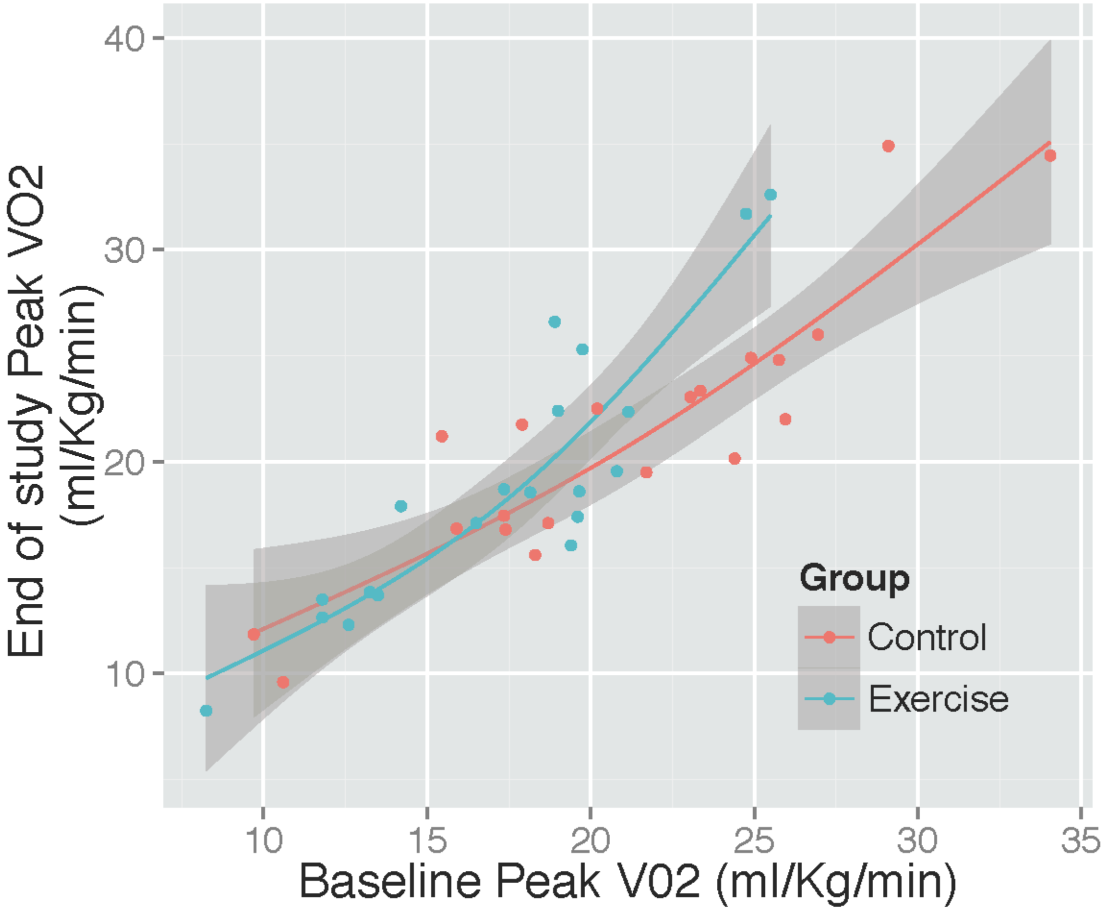

Supplement: Supplementary file 1 — Supplementary Material [file 41598_2017_18320_MOESM1_ESM.pdf]
